# From automatic integration to selective control: time-resolved effects of sensory cues on numerical comparison

**DOI:** 10.3389/fnins.2025.1660727

**Published:** 2025-10-08

**Authors:** Xiao Liang, Bo Jiang, Zonghao Zhang, Lijuan Wang

**Affiliations:** ^1^School of Educational Science, Shanxi University, Taiyuan, Shanxi, China; ^2^School of Modern Education, Changchun Guanghua University, Changchun, China; ^3^School of Education, Shanghai Jiao Tong University, Shanghai, China

**Keywords:** numerical comparison, approximate number system, sensory cues, congruency effect, sensory integration, inhibitory control, event-related potentials

## Abstract

**Introduction:**

Approximate numerical comparison is often influenced by various non-numerical sensory cues, yet whether they act via uniform inhibition (inhibitory control theory) or cue-weighted integration (sensory integration theory) remains debated.

**Methods:**

To clarify this theoretical controversy, the present study tested a cue-specific, temporally staged account by orthogonally manipulating numerosity with a holistic, highweight cue (convex hull) and a basic, lower-weight cue (average dot size) while recording fronto-central ERPs (P2, N450). Twenty-five adults performed a rapid dot array comparison under four congruency conditions.

**Results:**

Behavior showed clear convex-hull dominance: accuracy was high whenever convex hull aligned with numerosity and dropped when it conflicted, regardless of dot-size consistency; response times were unchanged. ERPs revealed a two-stage dynamic: the early P2 selectively tracked dot-size congruency (larger for dot-size–congruent trials), consistent with automatic integration of basic features, whereas the later N450 scaled with conflict structure and cue weight (fully congruent < dot-size–congruent < fully incongruent < convex-hull–congruent/dot-size–incongruent), with no latency differences.

**Discussion:**

These converging results support a time-resolved, weightsensitive mechanism in which basic features bias integration early and holistic configurations dominate later choice and recruit control when misaligned. The account reconciles sensory-integration and inhibitory-control views and motivates further tests of how cue–cue and cue–number conflicts shape numerical decisions.

## 1 Introduction

In everyday life, humans frequently need to make rapid numerical judgments and comparisons, such as selecting the shorter checkout line at a supermarket or determining which box of fruit offers more content for the same price (Feigenson et al., 2004; Halberda et al., 2008). This ability to estimate and compare quantities without precise counting primarily relies on the Approximate Number System (ANS), which supports the formation and comparison of imprecise numerical representations (Hyde and Spelke, 2011; Liang et al., 2022; Morín et al., 2025).

However, approximate numerical comparison is not based purely on abstract numerosity. A substantial literature shows that multiple non-numerical sensory cues—such as convex hull (the smallest region enclosing all items), average dot size (the mean area per item), total surface area, and density—reliably influence numerical judgments (Gebuis and Reynvoet, 2012a,b; Leibovich et al., 2017; Piazza et al., 2018; Tomlinson et al., 2020). When these cues covary with numerosity (e.g., the array with more dots also has a larger convex hull), accuracy is typically high; when they conflict with numerosity, accuracy can drop close to chance, a robust “congruency effect” (Clayton et al., 2015). This has fueled debate about how numerical representations interact with sensory cues.

Two prominent accounts offer different mechanistic emphases (Leibovich et al., 2017). The inhibitory control theory (also known as the Competing Processes Account) proposes that successful numerical judgments require active suppression of task-irrelevant or inconsistent sensory cues; by analogy with Stroop tasks, incongruent cues recruit additional control resources (Clayton and Gilmore, 2015), and individuals with stronger inhibitory control tend to show smaller congruency effects (Gilmore et al., 2013; Qi et al., 2025). In contrast, the sensory integration theory argues that numerical judgments emerge from the weighted integration of multiple cues without invoking a distinct inhibitory process; more diagnostic cues carry greater decision weight and thus bias choices (Gebuis and van der Smagt, 2011; Leibovich et al., 2017; Hofmann et al., 2024). Neuroimaging evidence indicates that non-numerical cues are processed even when numerosity is task-relevant, consistent with automatic integration (Gebuis and Reynvoet, 2013).

Although both accounts have empirical support and each explains aspects of how sensory cues relate to numerosity, they share a simplifying assumption that leaves the processing story incomplete: they tend to treat “sensory cues” as a homogeneous class and to assume a single-stage mechanism (Henik et al., 2017; Leibovich et al., 2017). The sensory-integration view emphasizes cue-weighted accumulation but underplays that a cue’s “weight” partly reflects its position in the visual hierarchy—basic features versus holistic configurations (Salti et al., 2017; Sanford et al., 2024). This hierarchical distinction has temporal implications: primary features are typically encoded more rapidly than higher-order configurations, so differences in cue weight are likely to map onto distinct encoding timelines and impose a temporal structure on integration itself (Burr and Ross, 2008; Piazza et al., 2018). Conversely, the inhibitory-control view highlights the need to override misleading cues but tends to under-specify two issues: high-weight cues often facilitate numerosity judgments when aligned, and the cost of overriding a cue plausibly scales with its weight rather than being uniform across cues (Clayton et al., 2015; Shilat et al., 2021; Sanford et al., 2024).

Taken together, these considerations motivate a cue-grounded complementarity: rapidly encoded basic features may support early, largely automatic, feature-based integration, whereas holistic configurations—typically computed later and suggested in prior work to receive greater decision weight—may exert a stronger influence on choice. When such configurations misalign with numerosity or other cues, they could elicit response competition and control recruitment, potentially beyond conflicts induced by basic features.

To make this complementarity testable, we targeted two cues that instantiate distinct computational and representational regimes: convex hull as a higher-weight holistic configuration, and average dot size as a basic lower-weight feature. We selected these cues because convex hull is a well-established dominant cue in approximate numerosity judgments, whereas average dot size is a tractable representative cue among strongly covarying alternatives (total surface area, density). This choice permits clean orthogonalization with numerosity and with each other, improving internal validity and allowing any temporal dissociation to be attributed to cue type rather than task demands (Salti et al., 2017; Sanford et al., 2024; Lorenzi et al., 2025). It also affords a principled link from computation to timing: basic features can support early feature-based integration, whereas holistic, higher-weight configurations are more likely to induce later conflict and control recruitment, yielding temporally dissociable signatures that single-stage accounts are less well positioned to explain.

Building on this cue selection, we combined behavioral measures with event-related potentials (ERPs) and orthogonally manipulated the congruency between numerosity and two non-numerical cues (convex hull, average dot size). This manipulation yielded four conditions that instantiate both cue-numerosity and cue-cue conflicts. Given this conflict-based design, the fronto-central midline could provide the most informative window, where early attentional selection and feature integration (fronto-central P2) and conflict monitoring and control recruitment (fronto-central N450) are reliably observed (Szűcs and Soltész, 2012; Gebuis et al., 2016). In contrast, occipito-parietal electrodes are typically leveraged to probe early perceptual stages of numerosity encoding (often via posterior components such as P2p; Hyde and Spelke, 2009, 2011; Fornaciai et al., 2017), which were not the target processes of the present study. We therefore adopted a hypothesis-driven fronto-central ROI and focused on two ERP components:

(1) The early P2 (approximately 200 *ms*) has been associated with perceptual attention and feature detection and is sensitive to basic visual properties in numerical tasks (Balconi and Carrera, 2011; Cohen Kadosh et al., 2011; Gebuis and Reynvoet, 2012a,b; Hyde and Spelke, 2011; Soltész and Szűcs, 2014).
(2) The later N450 (approximately 360–450 *ms*) has been linked to conflict processing and control recruitment in Stroop-like paradigms, with incongruent trials typically eliciting larger amplitudes than congruent trials (West, 2003; West et al., 2005; Larson et al., 2014; Liu et al., 2014). In numerical cognition, N450 modulations have likewise been observed when non-numerical cues conflict with numerical information (Szűcs and Soltész, 2012). In the present study, we therefore treat the N450 as an index of conflict monitoring and control engagement rather than as a direct readout of inhibition efficacy or error likelihood (e.g., Huang and Chen, 2020).

By jointly analyzing behavioral performance and the P2/N450 components under orthogonal manipulations of cue congruency, we ask whether early activity—particularly the P2—tracks congruency for a basic, lower-weight feature (average dot size) in a manner consistent with cue-weighted sensory integration, and whether later activity—the N450—scales with conflict when a higher-weight, holistic configuration (convex hull) is misaligned with numerosity or with another cue, as would be expected under selective recruitment of control.

## 2 Method

### 2.1 Participants

Twenty-seven undergraduate students (12 female, 13 male; age range = 19–23 years; *M* = 20.56, SD = 1.56) were recruited via campus advertisements. The target sample size was determined *a priori* using G*Power 3.1 (*α* = 0.05, *1 − β* = 0.80) for a within-subject medium effect (*Cohen’s d* = 0.65) based on prior ERP work using numerosity-cue congruency manipulations. Inclusion criteria were: normal or corrected-to-normal vision (visual acuity≥1.0), no color-vision deficiency; right-handedness (Edinburgh Handedness Inventory, LQ > 80); no history of neurological or psychiatric disorders or substance abuse; and no prior participation in similar numerical cognition tasks. Two participants were excluded due to excessive EEG artifacts (>30% trials), yielding a final sample of 25 participants (11 female; age = 20.48 ± 1.52 years). All participants provided written informed consent. The study protocol was approved by the local ethics committee and adhered to the Declaration of Helsinki.

### 2.2 Design

This study employed an approximate numerical comparison task where participants made quick judgments about which of two dot arrays contained more dots without counting (e.g., Gebuis et al., 2016; Inglis and Gilmore, 2013). The experiment followed a single-factor within-subjects design.

**Figure 1:**
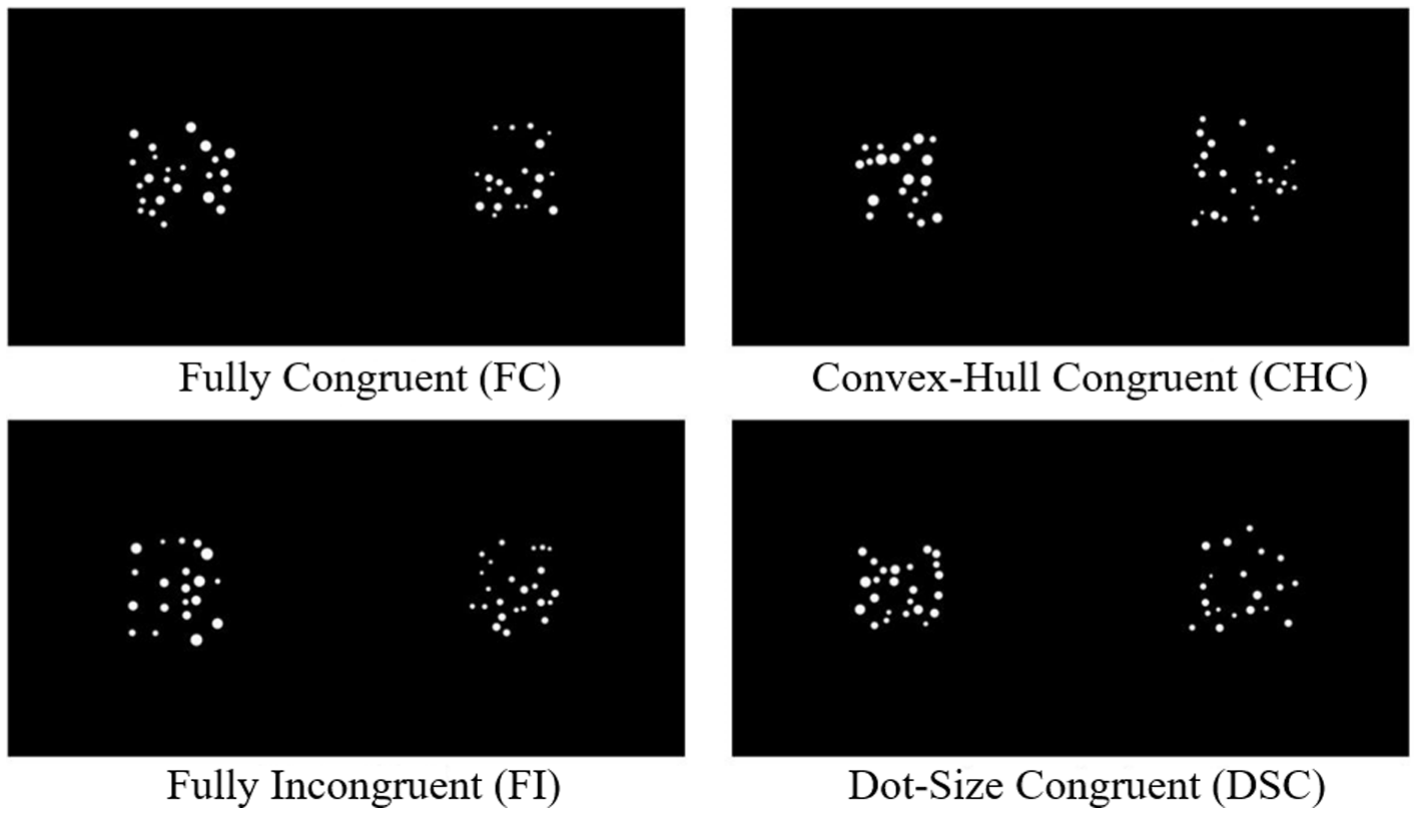
Four levels of congruency between sensory cues and numerical information.

The independent variable was the congruency between sensory cues and numerical information, comprising four levels: Fully Congruent (FC), Fully Incongruent (FI), Convex-Hull Congruent but average dot size incongruent (CHC), and Dot-Size Congruent but convex hull incongruent (DSC) (see Figure 1). In the FC condition, the numerically larger array featured both a larger convex hull and larger average dot sizes (diameters). In the FI condition, the numerically smaller array had both a larger convex hull and larger average dot sizes. In the CHC condition, the numerically larger array had a larger convex hull but smaller average dot sizes. In the DSC condition, the numerically larger array had larger average dot sizes but a smaller convex hull. The dependent variables included behavioral measures (accuracy and response time) and ERP amplitudes for P2 and N450 components, chosen *a priori* to index early feature-based processing and later control engagement, respectively, as motivated in the Introduction.

### 2.3 Materials

Stimuli were generated with a specialized MATLAB toolbox (De Marco and Cutini, 2020) that allows independent control of numerosity and non-numerical visual cues in dot arrays. Across trials, numerosity ratios were held near 1.2 (*Weber fraction* ≈ 0.2) using the pairs 18 vs. 22, 20 vs. 24, and 24 vs. 28, each equally represented across the four congruency conditions.

To instantiate the hypothesized dissociation between basic features and holistic configurations while maintaining internal validity, we orthogonally manipulated two cues—convex hull and average dot size—and balanced other image statistics that commonly covary with numerosity. Specifically: (1) Convex hull: the convex-hull ratio (larger/smaller) was matched across congruent and incongruent sets (*r* = 1.20 ± 0.10; range 1.05–1.45). Per-array hull area was approximately 50 ± 9 deg.^2^ (range 35–65 deg.^2^) across conditions. (2) Average dot size: individual dot diameters were sampled from non-overlapping ranges to produce perceptually distinct “small” (0.20°-0.30°) and “large” (0.40°-0.50°) sets while avoiding dot overlap within arrays. For each array, average dot size was defined as the mean dot diameter. (3) Balanced nuisance cues: total surface area, mean inter-dot distance, and global luminance were kept within narrow ranges across conditions (total surface area = 2.37 ± 1.30 deg.^2^; inter-dot distance = 0.95° ± 0.12°; display luminance = 124.1 ± 2.1 8-bit), minimizing unintended diagnostic information.

Arrays were displayed within two circular apertures (diameter = 10° of visual angle) centered at ±7.5° from fixation (center-to-center separation = 15°), leaving a 5° blank gap between inner edges. This geometry supported simultaneous perception of both arrays while discouraging saccades. Dots were white on a black background. Stimuli were presented on a 22-inch LCD monitor (1920 × 1,080, 60 Hz) at a 60 cm viewing distance; one pixel subtended ~0.024° of visual angle.

For reproducibility, we provide the MATLAB code used to generate the stimuli: https://doi.org/10.17605/OSF.IO/7RU2B.

### 2.4 Procedure

The experiment was implemented in E-Prime 3.0 and conducted in a sound-attenuated, electromagnetically shielded chamber. After providing informed consent, participants underwent head measurements and EEG setup, received standardized task instructions, and completed 20 practice trials (practice data were excluded from analyses).

**Figure 2:**
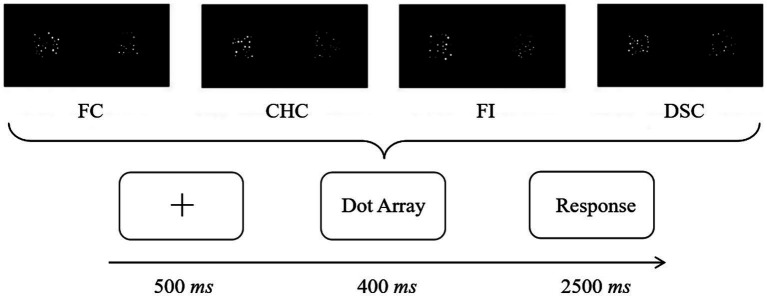
Experimental procedure.

Each trial comprised a central fixation cross (500 *ms*), simultaneous presentation of the two arrays (400 *ms*), and a response window on a blank screen until response was made or 2,500 *ms* elapsed (Figure 2). Participants were instructed to respond as quickly and accurately as possible while avoiding counting. The 400 *ms* presentation duration was chosen to minimize the opportunity for serial counting and encourage approximate judgments, consistent with prior work (e.g., Gebuis et al., 2016; Inglis and Gilmore, 2013). Responses were made with the index fingers resting lightly on the “F” and “J” keys (“F” = “more dots on the left,” “J” = “more dots on the right”) to minimize unnecessary movements and motor artifacts.

Each congruency condition contained 150 trials, for a total of 600 trials. Trials from the four conditions were randomly intermixed, and the side of the numerically larger array (left/right) was counterbalanced across the session. The experiment was divided into four blocks of 150 trials with self-paced breaks between blocks. Participants were encouraged to rest and blink during the breaks to maintain fixation quality during task blocks. The overall session, including setup and breaks, lasted approximately 60 min.

### 2.5 EEG recording

Electroencephalographic (EEG) was recorded with a Compumedics Neuroscan system (SynAmps 2 amplifiers) using a 64-channel Quik-Cap arranged according to the extended 10–20 system. AFz served as ground; the online reference was the left mastoid (M1). Vertical electrooculogram (VEOG) was recorded from electrodes above and below the left eye; horizontal electrooculogram (HEOG) from electrodes at the outer canthi. Electrode impedances were kept below 5 *kΩ*. Signals were digitized at 1000 *Hz* with an online band-pass of 0.01–40 *Hz* (24 dB/octave).

EEG preprocessing and artifact rejection were performed in EEGLAB (v2022.0; Delorme and Makeig, 2004). Data were re-referenced offline to the average of bilateral mastoids (M1 and M2). Continuous EEG was segmented from 1,000 *ms* before stimulus onset to 2000 *ms* after stimulus onset, with the 100 *ms* pre-stimulus interval serving as the baseline correction period. Independent Component Analysis (ICA) was applied together with ADJUST 1.1.1 (Mognon et al., 2011) and ICLabel (Pion-Tonachini et al., 2019) to identify and remove components reflecting ocular, muscular, and line-noise artifacts. Residual epochs exceeding ±100 *μV* or containing clear ocular deflections (as indicated by HEOG/VEOG or corresponding ICA components) were rejected.

Guided by the hypotheses and prior work linking fronto-central activity to feature integration and conflict monitoring in Stroop-like manipulations, we defined *a priori* a fronto-central ROI comprising FCz, FC1, and FC2. Two ERP components were quantified as mean amplitudes within canonical time windows: P2: 100–250 *ms* (peak ~200 *ms*), indexing early attentional/feature-related processing; N450: 250–500 *ms* (peak ~360 *ms*), indexing conflict monitoring/control engagement. Focusing analyses on midline fronto-central sites, which are topographically distinct from lateral sensorimotor areas, minimized overlap with response-related activity and reduced multiple-comparison burdens, thereby providing a direct test of the time-resolved predictions.

### 2.6 Data analysis

Behavioral and EEG preprocessing: For behavioral data, reaction times were computed on correct trials only; anticipations (<200 *ms*) and timeouts responses (>2,500 *ms*) were excluded a priori, resulting in an average trimming of approximately 2.8% per participant (about 17 trials). Accuracy was computed as the proportion correct per congruency condition after the same anticipations/timeouts were removed. For EEG, artifact correction combined ICA-based component removal with amplitude-based epoch rejection (±100 *μV*). On average, 8.6% of trials were excluded per participant during EEG preprocessing (51 trials). Because these behavioral and EEG exclusions partially overlapped, the final dataset for ERP averaging retained approximately 91% of all trials (about 546 of 600 trials per participant), i.e., an overall exclusion of roughly 9%.

To verify that trial exclusions did not disrupt balance across numerosity pairs, a repeated measures ANOVA on valid-trial counts across the Congruency × Numerosity-Pair cells revealed no significant main effect of Pair, F(2,48)=0.781,p=0.463, and no Congruency × Pair interaction, F(6,144)=0.854,p=0.535, indicating that the retained dataset remained pair-balanced across congruency conditions.

In formal analyses, we conducted one-way repeated-measures ANOVAs with four levels of congruency (FC, FI, CHC, DSC) separately for accuracy, reaction time, and mean amplitudes of each ERP component (P2, N450) at the priori fronto-central ROI (FCz, FC1, FC2). For visualization, grand-average ERP waveforms and scalp topographies were plotted at component-relevant latencies, and behavioral distributions were summarized with violin plots.

All data supporting the findings of this study are openly available at: https://doi.org/10.17605/OSF.IO/7RU2B.

## 3 Results

### 3.1 Behavioral results

First, we analyzed the effect of sensory cue congruency on accuracy (ACC) and reaction time (RT) in the approximate numerical comparison task.

**Figure 3:**
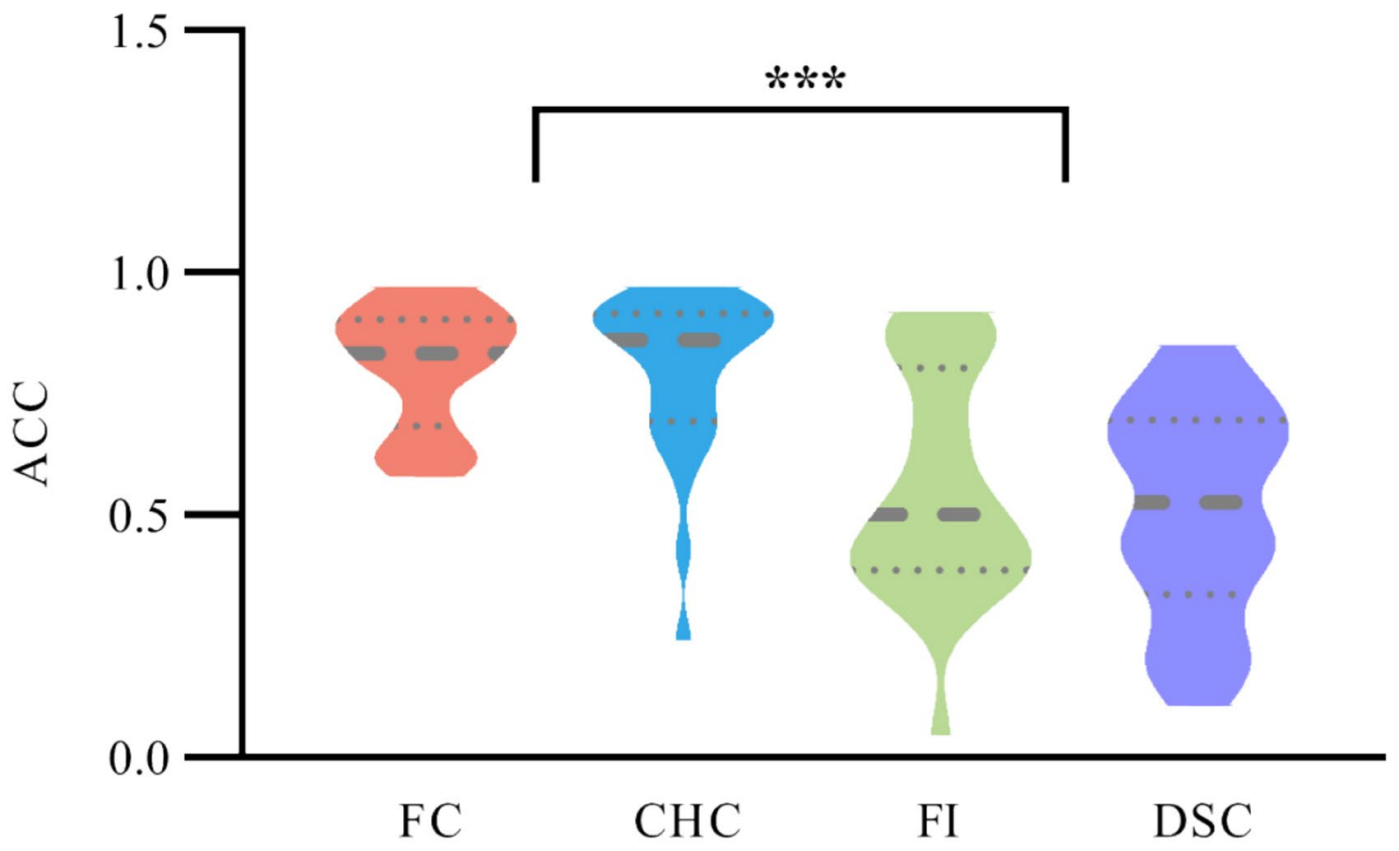
ACC across four congruency conditions. Violin plots depict the distribution of ACC; the horizontal thick solid line within each violin marks the median, and the thin dashed horizontal lines above and below mark the lower (25th percentile) and upper (75th percentile) quartiles—that is, the boundaries of the interquartile range. The violin outline indicates the kernel density of ACC values along the accuracy scale.

A repeated measures ANOVA on ACC across the four congruency conditions revealed a significant main effect of condition, $F(3,72)=17.934\;p<0.001,\eta_{p}^{2}=0.359$. Bonferroni post-hoc comparisons showed that participants’ ACC was significantly higher in the FC condition (M=0.82,SD=0.11) and CHC condition (M=0.81,SD=0.13) compared to the FI condition (M=0.63,SD=0.11,ps<0.001) and DSC condition (M=0.65,SD=0.10,ps<0.001). No significant differences were found between FC and CHC conditions or between FI and DSC conditions (ps > 0.05), as shown in Figure 3.

A repeated measures ANOVA on mean RT for correct trials showed no significant differences across the four congruency conditions, F(3,72)=0.192,p=0.903. RT in the FC condition (M=637,SD=112), FI condition (M=642,SD=115), CHC condition (M=635,SD=118), and DSC condition (M=640,SD=114) did not differ significantly.

### 3.2 ERP results

**Figure 4:**
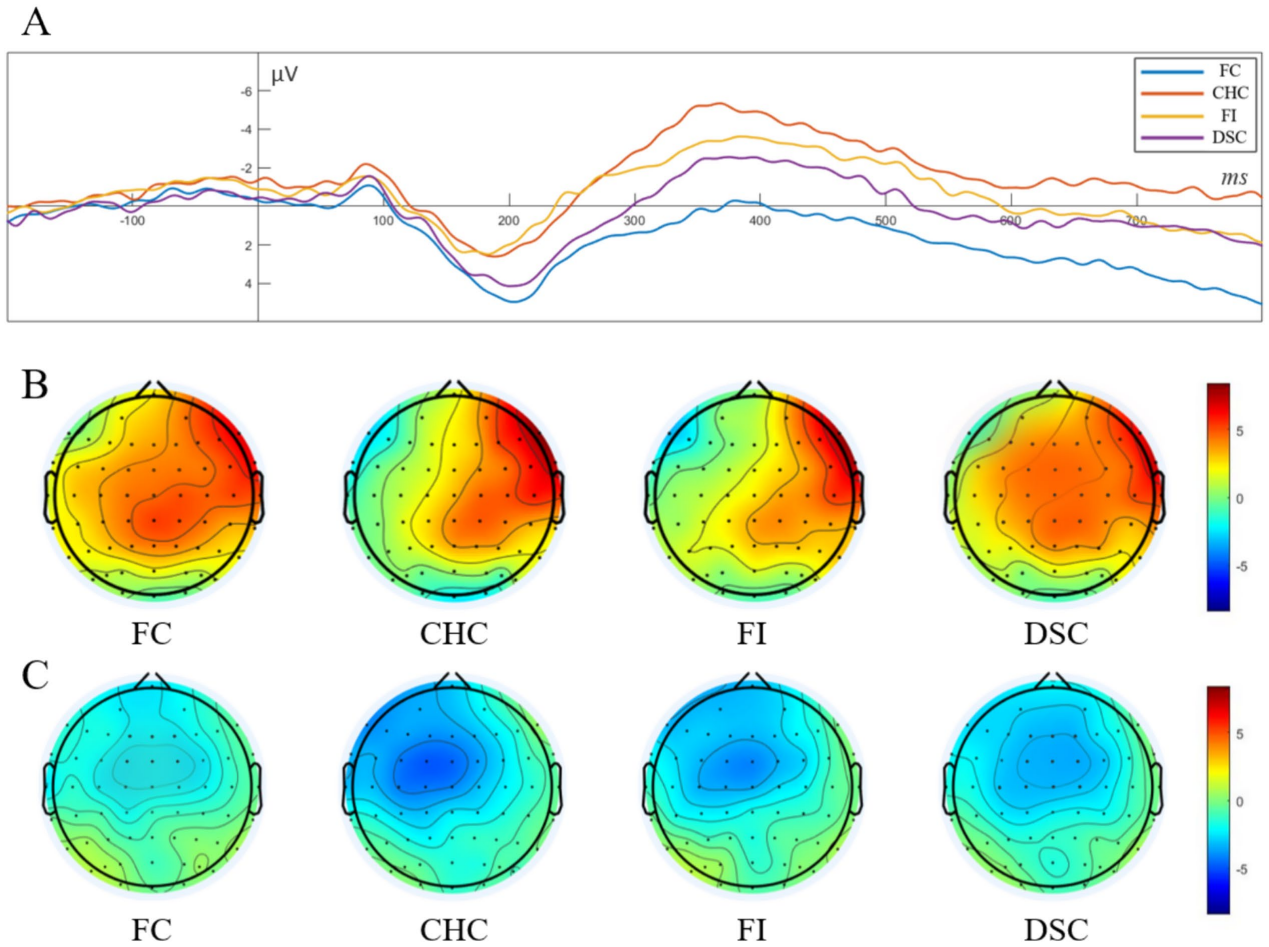
Grand-average ERP waveforms and scalp topographies for the four conditions. **(A)** Stimulus-locked waveforms from a fronto-central ROI (average of Fz, FCz, and Cz; baseline −100 to 0 *ms*; negative voltage plotted upward). Color code: FC (blue), CHC (red), FI (yellow), and DSC (purple). The components of interest are the P2 (100–250 *ms*; peak around 200 *ms*) and the N450 (250–500 *ms*; peak around 360 *ms*); **(B)** Scalp topographies of the mean voltage in the P2 window for each condition, showing a fronto-central positivity; **(C)** Scalp topographies of the mean voltage in the N450 window, showing a fronto-central negativity with the same graded amplitude pattern. Black dots mark electrode positions; color bars are in *μV* and share the same scale within each row.

A single-factor repeated measures ANOVA on P2 component amplitude revealed a significant main effect of congruency condition, $F(3,72)=30.247,p<0.001,\eta_{p}^{2}=0.285$. Post-hoc comparisons indicated that P2 amplitudes in the FC condition (M=4.95,SD=0.36) and DSC condition (M=4.04,SD=0.40) were significantly larger than those in the FI condition (M=2.03,SD=0.33) and CHC condition (M=2.37,SD=0.36), ps<0.001. No significant differences were observed between FC and DSC conditions or between FI and CHC conditions (ps>0.05). In other words, P2 amplitudes from smallest to largest followed the pattern: FI = CHC < DSC = FC. See Figure 4A for waveforms, Figure 4B for P2 topographical maps.

To further assess whether variation in numerosity pairs across congruency conditions could bias P2, a repeated measures ANOVA on P2 amplitude stratified by numerosity pair (18–22, 20–24, 24–28) across the four congruency conditions found no significant main effect of Pair, F(2,48)=0.625,p=0.547, and no Pair × Congruency interaction, F(6,144)=0.782,p=0.598, indicating that P2 amplitude differences were not driven by the total number of dots on the screen.

A single-factor repeated measures ANOVA on N450 component amplitude demonstrated a significant main effect of congruency condition, $F(3,72)=54.843,p<0.001,\eta_{p}^{2}=0.426$. Post-hoc comparisons showed that the CHC condition (M=−5.34,SD=0.55) elicited the largest negative amplitude, which was significantly greater than those in the FI condition (M=−3.52,SD=0.49) and DSC condition (M=−2.71,SD=0.42), ps<0.001. The negative amplitude in the FI condition was also significantly larger than those in both the DSC (p=0.045<0.05) and FC conditions (M=0.11,SD=0.24,p<0.001). Additionally, the amplitude in the DSC condition was significantly larger than that in the FC condition (p<0.001). In summary, N450 amplitudes from smallest to largest followed the pattern: FC < DSC < FI < CHC. See Figure 4C for N450 topographical maps.

To characterize the temporal properties of these components across congruency conditions, we also examined peak latency. For each participant and condition, peak latency was extracted within the P2 (100–250 *ms*) and N450 (250–500 *ms*) windows and submitted to single-factor repeated measures ANOVAs. Neither analysis revealed a significant main effect of congruency: P2 latency, F(3,72)=0.925,p=0.447; N450 latency, F(3,72)=1.186,p=0.323, indicating that congruency effects were expressed in component amplitude rather than in peak timing.

## 4 Discussion

This study tested whether non-numerical cues influence numerical comparison through a single homogeneous pathway or through cue-specific, temporally staged processes. By varying numerosity with a holistic, high-weight cue (convex hull) and a basic, lower-weight cue (average dot size) and targeting fronto-central ERPs, we found a clear temporal and functional dissociation. Behavior tracked convex-hull congruency (accuracy: FC=CHC > FI=DSC; RTs unchanged), the early P2 indexed dot-size congruency (FI=CHC < DSC=FC), and the later N450 scaled with cross-cue conflict and peaked when convex hull supported numerosity but average dot size opposed it (FC < DSC < FI < CHC). Together, these findings reveal staged processing—early automatic, feature-based integration followed by later conflict monitoring and control recruitment—and bridge sensory-integration and inhibition accounts by showing that cue weights and their entry times jointly shape numerical decisions.

### 4.1 Convex hull dominance without speed costs

The accuracy pattern provides clear behavioral evidence for differential cue weighting in approximate numerical comparison. When convex hull was congruent with numerosity (FC, CHC), accuracy was high; when convex hull was incongruent (FI, DSC), accuracy dropped markedly, irrespective of dot-size congruency (FC=CHC > FI=DSC). This asymmetry is consistent with sensory-integration accounts in which cues contribute to choice in proportion to their diagnostic value or reliability (Gebuis and Reynvoet, 2012a,b; Leibovich et al., 2017). As a holistic descriptor of the spatial extent of the array, convex hull appears to carry greater decision weight than average dot size and thus exerts a dominant influence on judgments (Sanford et al., 2024; Shilat et al., 2021). Notably, the fact that dot-size congruency failed to rescue performance when convex hull opposed numerosity (DSC) further supports the idea that low-weight features have limited leverage over choice when high-weight, configuration-level cues point the other way.

The contrast between CHC and DSC offers additional insight into how cue weights may shape control recruitments. When the higher-weight cue (convex hull) aligns with numerosity (CHC), performance remains strong, suggesting that any interference from the lower-weight feature (average dot size) is comparatively easy to manage. By contrast, when the higher-weight cue misaligns (DSC), accuracy declines even though the lower-weight cue favors the correct response, consistent with the notion that overriding a high-weight distractor is harder than suppressing a low-weight one (Clayton and Gilmore, 2015; Gaspelin and Luck, 2018; Leong et al., 2017; Theeuwes, 2010). Formally, the Expected Value of Control framework (Shenhav et al., 2013, 2017) provides a useful normative lens: control intensity should scale with the anticipated benefit of exerting control, which depends on both payoff and the probability of successful override. In our task, CHC likely affords higher expected benefit (suppressing a low-weight distractor is more likely to succeed), consistent with higher accuracy; DSC affords lower expected benefit (overriding a high-weight distractor is less likely), consistent with reduced accuracy. Nevertheless, behavioral data alone cannot establish weight-dependent control; we return to this question using ERP evidence to probe when and how control is engaged (see Section 4.2).

Response times did not differ significantly across conditions, indicating that the convex-hull dominance effect primarily manifested in decision quality rather than decisional speed under our brief (400 *ms*) simultaneous displays. In relation to Shilat et al. (2021), both studies converge on a robust role for convex hull in numerical judgments. Our null RT differences may reflect several design choices that limit strategic slowing and serial sampling: orthogonalized cue manipulations, tightly matched numerosity ratios (about 1.2), randomly intermixed conditions, and a short exposure intended to discourage counting. It is also possible that RT was less sensitive than accuracy to cue-driven interference at this difficulty level or that our study was powered to detect accuracy but not modest RT effects. Future work combining formal decision modeling (e.g., drift–diffusion) with manipulations of presentation duration and cue strength could clarify whether convex hull primarily reduces evidence quality (drift) versus alters decision thresholds, thereby clarifying when cue weighting impacts speed in addition to accuracy.

### 4.2 Time-resolved integration and conflict monitoring

The ERP pattern reveals a staged influence of sensory cues on numerical comparison that aligns with, yet refines, a cue-weighted integration account. Early activity (P2, ~200 ms) selectively tracked dot-size congruency, whereas later activity (N450, ~360–450 ms) scaled with the structure of conflict across cues and with numerosity.

Early stage: feature-driven sensitivity. At the fronto-central ROI, P2 amplitudes were larger when average dot size supported numerosity (FC, DSC) and smaller when average dot size opposed it (FI, CHC), independent of convex-hull congruency (FI=CHC < DSC=FC). This suggests that basic features exert an early, largely automatic influence on processing, consistent with rapid extraction of low-level attributes and their early entry into the decision process (Salti et al., 2017; Sanford et al., 2024). Importantly, stratifying by numerosity pair ruled out trivial explanations based on the total number of dots on the screen. We are cautious not to claim that P2 reads out average dot size per se; rather, in the present design, dot-size congruency appears to be the dominant determinant of early attentional/feature-related activity. This aligns with prior findings of P2 sensitivity to basic visual properties and with the expectation that basic features are encoded earlier than holistic configurations (Burr and Ross, 2008; Hyde and Spelke, 2011).

Later stage: graded conflict monitoring and control recruitment shaped by cue weight and alignment. The N450 showed a clear gradient (FC < DSC < FI < CHC): minimal conflict when all cues aligned; increasing engagement with misalignments; and maximal engagement when average dot size opposed numerosity while convex hull supported it (CHC). To interpret this asymmetry, we separate two partially dissociable sources of tension: cue-cue conflict (C-C; when convex hull and average dot size favor opposite responses, present in CHC and DSC) and cue-numerosity conflict (C–N; when sensory cues misalign with numerosity; partial in CHC and DSC, strongest in FI, absent in FC), together with an asymmetry in cue weights (convex hull > average dot size).

Within this conflict structure, the CHC condition jointly presents cue-cue conflict and partial cue-numerosity conflict but benefits from the high-weight cue (convex hull) aligning with the goal. Under these circumstances, an early feature-driven bias (from average dot size) must be overridden later by the higher-weight configuration and the task rule, yielding strong conflict monitoring and control signals (largest N450) while accuracy remains high. By contrast, DSC also contains cue-cue and partial cue-numerosity conflict, but here the high-weight cue misleads. If overriding a dominant but misleading cue is relatively unlikely to succeed, control engagement may be attenuated compared with CHC, producing a smaller N450 alongside reduced accuracy. Finally, FI lacks cue-cue conflict but imposes the strongest cue-numerosity conflict because both cues mislead; this elicits substantial—yet not maximal—N450 and low accuracy.

Framed within the Expected Value of Control perspective (Shenhav et al., 2013, 2017), these differences can be understood as reflecting the anticipated benefit and efficacy of exerting control given cue weights and alignment: when success is likely (overriding a low-weight distractor; CHC), control is strongly recruited; when success is doubtful (overriding a high-weight distractor; DSC) or when no internal cue-cue tension enforces a competing alternative (FI), control signals are comparatively smaller. We emphasize that this is a principled interpretation rather than a definitive mechanistic claim; single-trial and model-based analyses would be needed to arbitrate among alternatives.

Two additional observations qualify this account. First, neither P2 nor N450 peak latency differed across conditions, suggesting that congruency primarily modulated the strength, not the timing, of early feature processing and later conflict monitoring. Second, the neural-behavioral asymmetry—maximal N450 in CHC despite high accuracy—underscores that N450 indexes conflict monitoring and control engagement rather than inhibition success or error likelihood per se, consistent with its role in Stroop-like tasks (e.g., Liu et al., 2014; Szűcs and Soltész, 2012).

Taken together with the behavioral dominance of convex hull (Section 4.1), the ERP results support a cue-grounded complementarity: basic features shape early processing (P2), whereas holistic, higher-weight configurations dominate choice and recruit control when misaligned (N450). This time-resolved view integrates sensory-integration and inhibitory-control perspectives by linking cue weights to their likely order of availability and to the cost of resolving conflicts among cues and with the task goal.

In sum, we outline a time-resolved, weight-sensitive account: basic features enter integration earlier (indexed by P2), whereas holistic configurations dominate later choice and recruit conflict monitoring when misaligned (indexed by N450). These mechanisms are complementary and yield testable predictions that call for single-trial and model-based validation. Specifically, to strengthen and refine this account, future work can use single-trial regression to parse N450 into contributions from cue–cue conflict, cue–number conflict, and the alignment of the higher-weight cue with the goal and relate these to trial-by-trial accuracy; manipulate presentation duration or feature-onset asynchrony to test the “speed-of-availability” hypothesis; and integrate ERP with decision models (e.g., drift-diffusion with time-varying drift weights) to link P2 and N450 to distinct computational stages.

### 4.3 Significance, limitations, and future directions

The main contribution is a time-resolved, weight-sensitive account that reconciles sensory-integration and inhibitory-control perspectives: cues of different weights enter and influence the decision process in an ordered fashion, with misalignment recruiting conflict monitoring/control. Grounded in operational neural markers, this account outlines a bridge between normative control theories and evidence-accumulation models in multi-cue, single-decision contexts, and yields testable predictions (e.g., temporal manipulations and single-trial modeling of availability and reweighting). Potential implications for education or intervention (e.g., vigilance to high-weight but potentially misleading cues and selective control) remain preliminary and call for rigorous cross-task and cross-population validation.

Notwithstanding these contributions, several limitations point to clear avenues for future work. First, we orthogonally manipulated only two cues (convex hull and average dot size), and broader mapping across density, total area, connectivity, and spatial arrangement is needed to assess the generality and interactions of cue weights. Second, the numerosity ratio (~1.2) and exposure duration (400 ms) were relatively fixed, and null effects on RT and peak latencies may partly reflect constraints of task parameters and statistical power; larger samples, latency-sensitive methods (e.g., jackknife, fractional-area, single-trial estimates), and parametric manipulations of cue strength, signal-to-noise ratio, and exposure duration can directly test the “speed-of-availability” hypothesis. Third, our analyses focused on a fronto-central ROI and scalp-level amplitudes without systematically probing posterior components or broader spatial distributions; future work should include occipito-parietal regions and posterior components (e.g., P2p) to examine hierarchical division of labor and assess or control motor-preparation influences (e.g., LRP) to rule out response-related confounds. Finally, our analyses were confined to scalp-level amplitudes and did not include source reconstruction; with a 64-channel montage and no individual structural MRIs, any inverse solution would be exploratory with limited spatial precision. To improve spatial specificity, future studies should use high-density EEG/MEG with subject-specific head models and, where feasible, multimodal integration (EEG–fMRI/MEG).

## 5 Conclusion

Approximate number comparison is jointly shaped by cue weights and their temporal order of availability: basic features influence evidence integration earlier and more automatically, whereas holistic configurations dominate later choice and trigger conflict monitoring and control when misaligned. Converging behavioral and neural evidence supports this time-resolved, weight-sensitive account, offering a concise integration of sensory-integration and inhibitory-control views and yielding testable predictions—via temporal manipulations and single-trial modeling—to further evaluate its generality and mechanisms.

## Data Availability

The datasets presented in this study can be found in online repositories. The names of the repository/repositories and accession number(s) can be found in the article/supplementary material.
